# Increased sensitivity of heavy metal bioreporters in transporter deficient *Synechocystis* PCC6803 mutants

**DOI:** 10.1371/journal.pone.0261135

**Published:** 2021-12-16

**Authors:** Gábor Patyi, Barbara Hódi, Dániel Solymosi, Imre Vass, Péter B. Kós

**Affiliations:** 1 Institute of Plant Biology, Biological Research Centre of the Eötvös Lóránd Research Network, Szeged, Hungary; 2 Faculty of Science and Informatics, Doctoral School in Biology, University of Szeged, Szeged, Hungary; 3 Department of Biotechnology, Faculty of Science and Informatics, Szeged University, Szeged, Hungary; Zhengzhou University, CHINA

## Abstract

The detection and identification of heavy metal contaminants are becoming increasingly important as environmental pollution causes an ever-increasing health hazard in the last decades. Bacterial heavy metal reporters, which constitute an environmentally friendly and cheap approach, offer great help in this process. Although their application has great potential in the detection of heavy metal contamination, their sensitivity still needs to be improved. In this study, we describe a simple molecular biology approach to improve the sensitivity of bacterial heavy metal biosensors. The constructs are luxAB marker genes regulated by the promoters of heavy metal exporter genes. We constructed a mutant strain lacking the cluster of genes responsible for heavy metal transport and hence achieved increased intracellular heavy metal content of the *Synechocystis* PCC6803 cyanobacterium. Taking advantage of this increased intracellular heavy metal concentration the Ni^2+^; Co^2+^ and Zn^2+^ detection limits of the constructs were three to tenfold decreased compared to the sensitivity of the same constructs in the wild-type cyanobacterium.

## Introduction

The continuous accumulation of heavy metals (HMs) is a common environmental phenomenon in aquatic and terrestrial ecosystems through human activities, and this pollution is persistent because these compounds cannot be degraded hence they are accumulated by organisms [1, 2]. The effluents produced by the processing industry contain a variety of heavy metals (HM), such as nickel, cobalt, zinc, copper, and cadmium. These compounds significantly contribute to the increase of toxic HM pollution in the environment. In addition to industrial pollution, traffic-related street dust has also become increasingly important in recent years, which can cause an extremely high health risk in big cities [3]. A connection was found between inhalable cobalt and respiratory symptoms and lung dysfunctions in the Swedish hard metal industry [4]. The average inhalable cobalt concentration was 0.0017 mg/m^3^. In roadside samples, the Co^2+^ level of the soil samples ranged from 1.9 to 3.5 mg/kg [5]. The amount of zinc and nickel contamination is more significant in rivers and lakes. In Europe, reported nickel concentrations in drinking water were generally below 10 μg L^-1^ (0.17 μM). The levels of zinc in surface water and groundwater normally do not exceed 0.01 and 0.05 mg L^-1^ (0.05–0.25 μM) [6]. According to a study from 2020 in India, the concentration of cobalt was about 0.003 μM, nickel 0.22 μM, and zinc up to 0.82 μM, respectively, in the studied river area [7].

Due to its importance, the detection and removal of toxic heavy metals have increased attention, and various bioremediation materials, such as plants, fungi, and bacteria have been employed [8–10].

Cyanobacteria are present in both aquatic and terrestrial ecosystems. Particularly they can be major primary producers in oligotrophic and freshwater systems. Cyanobacteria are photosynthetic gram-negative prokaryotes; they are the only known prokaryotes that perform oxygen-evolving photosynthesis. Furthermore, cyanobacteria require a variety of metal cations such as Cu^2+^, Ni^2+^, Fe^2+^, and Zn^2+^ to maintain their cellular metabolism and growth [11–13]. Besides requiring high amounts of various metals for living, cyanobacteria are also frequently affected by drastic changes in metal concentrations and are often challenged by heavy metal toxicities. To survive in the polluted environment a sensitive regulation system in metal uptake and removal is required. Cyanobacteria have complex metal resistance system mechanisms including exclusion via active transport by ATPases or chemiosmotic efflux systems. These P-type ATPases transport metal ions from the cytosol into the periplasm [14]. Our knowledge of the cyanobacterial heavy metal regulation system is always increasing as new metabolic pathways are discovered. However, the systematization and collection of this information is not easy, since the metal tolerance and requirement are different in almost every species. The cells have to acquire the correct element for each cellular function, but different metal ions can compete for the same binding site [15].

The cyanobacterium *Synechocystis* sp. PCC6803 (hereafter referred to as *Synechocystis*) has a small well-known genome, and it is easy to manipulate, hence it receives strong attention in basic and applied research. In the *Synechocystis* genome, a metal regulated gene cluster was identified, which is involved in the heavy metal resistance via export of zinc, cobalt, and nickel [16]. This cluster is a 12 kb long region and includes 11 open reading frames (ORFs) consisting of six putative transcriptional units as follows: the *coaT* and *coaR* involved in the Co^2+^ tolerance the *nrsBACD* operon involved in the Ni^2+^ and Co^2+^ homeostasis and regulated by the *nrsRS* operon region, a hypothetical membrane protein-coding gene and the *ziaA* and *ziaR* involved in the Zn^2+^ tolerance [17].

As we showed earlier [18] the transcriptional repressors are rather sensitive to the corresponding heavy metals, hence the above-mentioned transport proteins are upregulated at low heavy metal concentrations, where general stress responses are not triggered. The observation enabled us to construct whole-cell biosensors in *Synechocystis* by fusing the *coaT* and *nrsBACD* promoters with the reporter genes *luxAB* encoding easily detectable luminescent proteins [19]. These strains can sense the metal ions present in their environment, representing an alternative to traditional analytical chemical methods, with the advantage to detect the bioavailable fraction (rather than total concentration) of an analyte, allowing for a more accurate assessment of the biological significance of the pollution. These *nrsLux* and *coaLux* biosensors responded to the respective heavy metal ions. After 3 hours of incubation in the case of *coaLux* reporter the detection range was 0,3–6 μM Co^2+^ and 1–3 μM Zn^2+^ and the *nrsLux* strain was specific in the range of 0,2–6 μM Ni^2+^ concentration [19].

In the current study, we aimed to increase the sensitivity of the biosensor strains. To this end, we constructed a knock-out *ΔnrsRSBACD*:*ΔcoaRT*:*ΔziaRA* mutant strain, which then lacks the above described heavy-metal responsive gene cluster coding for the export of the given heavy metal ions; hence the ratio of intracellular vs. extracellular heavy metal concentration was expected to be higher than that in the wild type. For easy readability and pronunciation, we designated the knockout mutant as NiCoZia strain, so this designation will be used hereafter. We created the *nrsR*, *coaR*, and *ziaR* promoter-driven heavy metal responsive luminescent biosensors both in wild type *Synechocystis* and in this mutant cell line. We found a significant increase of intracellular heavy metal concentration in the mutant that resulted in up to a tenfold increase in sensitivity of the reporters.

## Materials and methods

### Strains, growth conditions, and heavy metal salt treatment

*Synechocystis* cells were grown in photoautotrophic 3% CO_2_-rich atmospheric condition under 40 μmol photon m^−2^ s^−1^ white light intensity and 30°C in BG-11 [20] liquid medium. For the preparation of the starter culture, 1.5 mL frozen stock culture was inoculated to 50 mL liquid BG-11 medium containing the appropriate antibiotic. From the fully-grown starter culture, 1 mL was inoculated into 200 mL BG-11 without antibiotics and incubated until logarithmic phase (OD_720nm_: 0.8). Heavy metal salt stress treatments were carried out in test tubes with the appropriate (ZnSO_4_, CoCl_2_, NiCl_2_) salt supplemented BG-11 liquid media without glucose. For the segregation of *ΔnrsRSBACD*:*ΔcoaRT*:*ΔziaRA* (NiCoZia) strain 50 μg mL^-1^ spectinomycin (Spe), and for the biosensor constructs 50 μg/ml kanamycin (Km) were used. The BG-11 medium contains 0,137 μM Co^2+^ and 0.77 μM Zn^2+^.

*Escherichia coli* strain DH5α was used for routine DNA manipulations [21] and constructions in Luria broth (LB) medium at 37°C [22]

### Construction of NiCoZia strain

**Fig 1:**
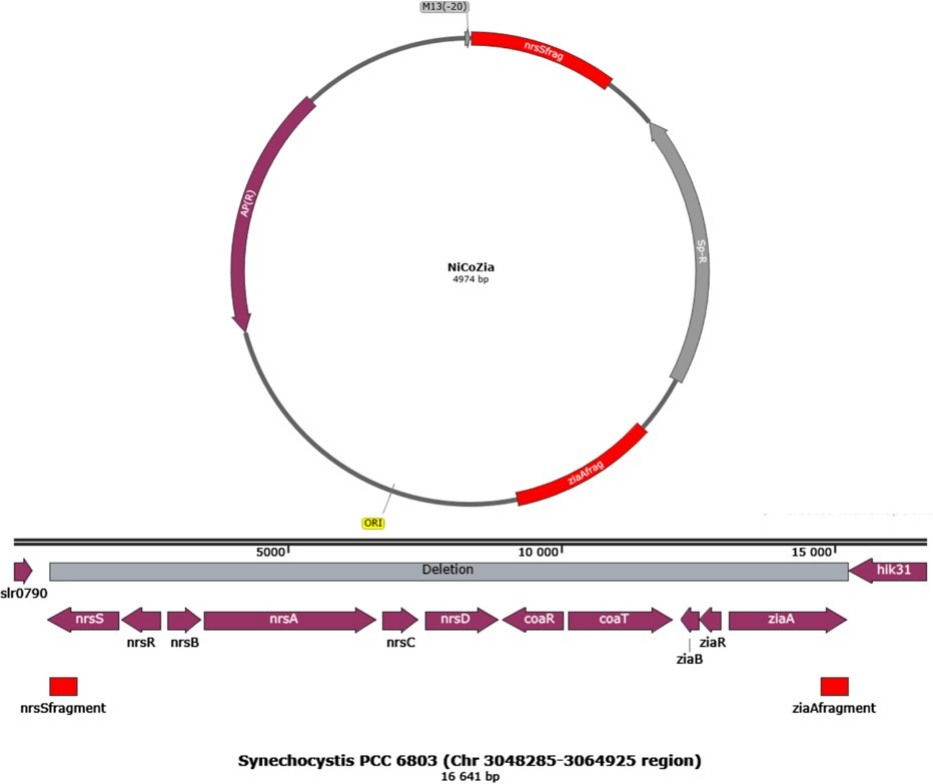
Schematic representation of the cloning strategy used for construction of the NiCoZia strain. The upper part of the figure shows the plasmid map of the pUC19 with the nrs-Spe^R^-zia fragment. The lower part of the figure shows the construction of the heavy metal cluster and the homologous regions.

We constructed a mutant where the gene cluster was replaced by a spectinomycin cassette (Sp-R). An insert was built up into pUC19 vector from PCR amplified fragments with flanking restriction sites of the distal ends of *ziaR* (PstI, XbaI), *nrsS* (KpnI, EcoR) using *Synechocystis* genome DNA and the spectinomycin cassette (XbaI, KpnI) using the vector pDF-trc [23] as the template (Fig 1). The plasmid was amplified in *E*. *coli* and transformed to *Synechocystis* via natural transformation. The mutant formed by double crossover was grown on a selective BG-11 plate containing 50 μg mL^-1^ spectinomycin (Spe).

### Construction of bioreporter strains in WT and NiCoZia

**Fig 2:**
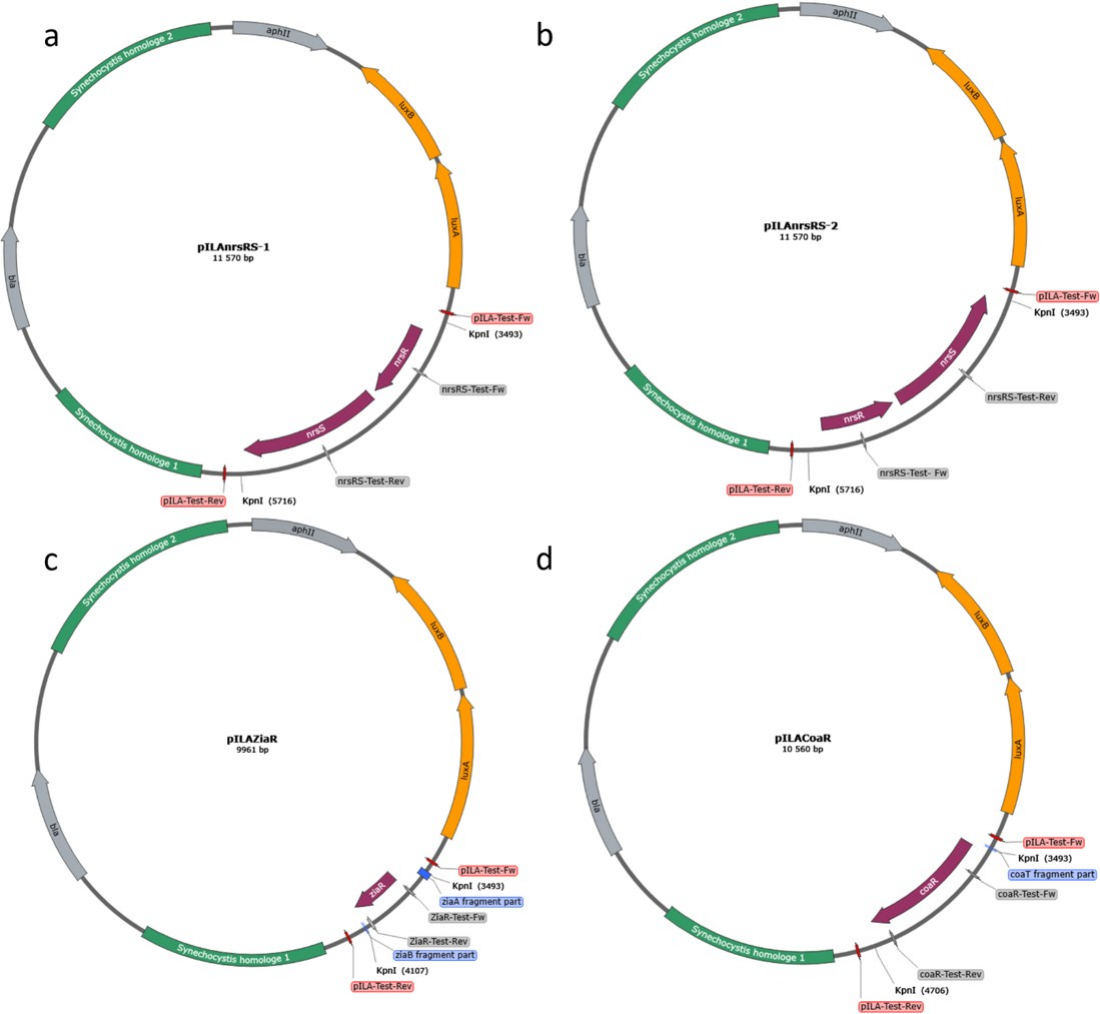
Schematic figure of the promoter probe vectors. Schematic figure of the promoter probe vector pILA with the inserts of *coaR* (d), *ziaR* (c), and with the two differently oriented *nrsRS* (a; b). We used the appropriate primers (Table 1) for the fragments’ amplification from the WT *Synechocystis* genome and applied KpnI digestion. *LuxA* and *LuxB* genes code for the Luciferase reporter proteins, which are required for the detection. The *aphII* and *bla* refer to genes conferring resistance to ampicillin and kanamycin, respectively.

We used the pILA promoter probe vector [24] utilizing the *LuxAB* luminescence reporter system. The insert of the *coaR; ziaR* and *nrsS + nrsR* (in short: *nrsRS*) promoter region was amplified by PCR using chromosomal DNA of WT *Synechocystis* PCC6803 as a template and the appropriate primer pair with a KpnI flanking at the 5’ end of both primers (Table 1) The promoter fragments were inserted into the unique KpnI site of pILA. Transformants of *E*. *coli* with the right orientation were selected via colony PCR using test primer pairs. The newly created pILAcoaR, pILAziaR and pILAnrsRS-1 and pILAnrsRS-2 plasmids (Fig 2) were amplified and isolated from *E*. *coli*. Wild type and NiCoZia *Synechocystis* strains were transformed and the clones were selected with Km selection as described above.

**Table 1:** Oligonucleotides used in this work.

Gene symbol	Oligonucleotide sequence
nrsS-Frg-Fw	5’TTGAATTCGTAACTGGATGGTGAATAAACTTCCCTT
nrsS-Frg-Rev	5’TTGGTACCTACGGATTTATTGCTACTAAGTCGCTTA
ziaA-Frg-Fw	5’TTTCTAGAGAGACCTAAAACGCATGGGAGTTGAAAA
ziaA-Frg-Rev	5’TTCTGCAGCTTAGCAATCCGAGTAGCATTCAAAATC
SpecR-XbaI	5’TTTCTAGACCGGAGACGGTCACAGCTTGTCTGTAAG
SpecR-KpnI	5’TTGGTACCATGTATGCTCTTCTGCTCCTGCCGGCCGA
CoaR-pILA-Fw	5’AAAAAGGTACCTTCACCATCCTTTCCCTATC
CoaR-pILA-Rev	5’AAAAAGGTACCACCTTCTCAGCCTAAACC
CoaR-Test-Fw	5’CAGGGCTTTCAGTTGTCT
CoaR-Test-Rev	5’GGTGATATGGGGAATGGG
NrsRS-pILA-Fw	5’AAAAAGGTACCGACGGCGTAAAGTTGATAAA
NrsRS-pILA-Rev	5’AAAAAGGTACCTCCCCCGCTAAGATCAGA
NrsRS -Test-Fw	5’TATTAGCAAGACTGCGGG
NrsRS-Test-Rev	5’TGTTGTTGTTGTTGGTAGG
ZiaR-pILA-Fw	5’AAAAAGGTACCCATCGTCCATCTCCTTAATC
ZiaA-pILA-Rev	5’AAAAAGGTACCCCGACTTGCATTTGCTGA
ZiaR-Test-Fw	5’TCCTAACGCCAACCTCTA
ZiaR-Test-Rev	5’CCCGATACAAATTCATCACA
pILA-Test-Fw	5’ACAACCAAATTTTCCCCAAG
pILA-Test-Rev	5’TCGATAGTGGCTCCAAGT

### Bioluminescence assay

Heavy metal salt treatments were carried out in 96 well black (Opti-Plate) cell culture plates with low autofluorescence (Perkin-Elmer) in 25°C and 40 μE light intensity. Each well contained 200 μL of cell culture (OD_720_: 0,8–1) and 100 μL of salt solution in different concentrations. The plates were covered with punctured transparent foil [19]. After the 3-hour long treatment added 6 μL of 50 mM decanal dissolved in 50% Methanol to 300 μL of the samples placed per well. Luminescence of the luciferase reaction is induced by the addition of the decanal to the 300 μL cultures in 1 mM final concentration [24]. After the addition of the substrate, the samples were preincubated in dark for 2 minutes before the light emission monitoring. Assays were performed with four parallel samples at room temperature. Luminescence was determined by a Top Count NXT luminometer (Packard Instruments), with the expression of counts per second (CPS) and normalized to the OD_720_ values of the cells.

### Measurement of growth inhibition

The differences in growth intensity caused by excess metal ions in WT and NiCoZia cyanobacterial cultures were quantified by measuring the optical density at 680 nm and 720 nm in 30μE light intensity and 30°C temperature for a period of 2 to 4 days. We set the starter *Synechocystis* culture’s optical density to OD_720_ = 0.1 and added heavy metal salt in 8 different concentrations (0 μM; 0.05 μM; 0.1 μM; 0.25 μM; 0.5 μM; 1 μM; 2.5 μM; 5 μM) in 50 mL final volume. For the measurement, we used a Photo Multi Cultivator MC-1000 (Photon Systems Instrument) with automatic OD measurement every hour. Two inhibition parameters were determined for each metal ion, minimal inhibitory concentration (IC_min_) and maximal inhibitory concentration (IC_max_). The IC_min_ refers to the lowest tested concentration leading to growth inhibition and IC_max_ refers to the highest tested concentration where no further growth was observed.

### Determination of the intracellular heavy metal content

*Synechocystis* cultures were supplemented with 5 μM of the respective salts. Cells were collected after 3-hour incubation by centrifugation (6000 rpm 10 min), washed once with BG-11 solution, and freeze-dried. From 50 mL cultures 40 ± 5 mg wet weight cell pellet could be harvested, from which 5 ± 1 mg dry material could be obtained. The heavy metal content of the samples was determined as described earlier [18].

## Results

### Growth inhibition in the NiCoZia (*ΔnrsRSBACD*:*ΔcoaRT*:*ΔziaAR)* and the wild type strain

**Fig 3:**
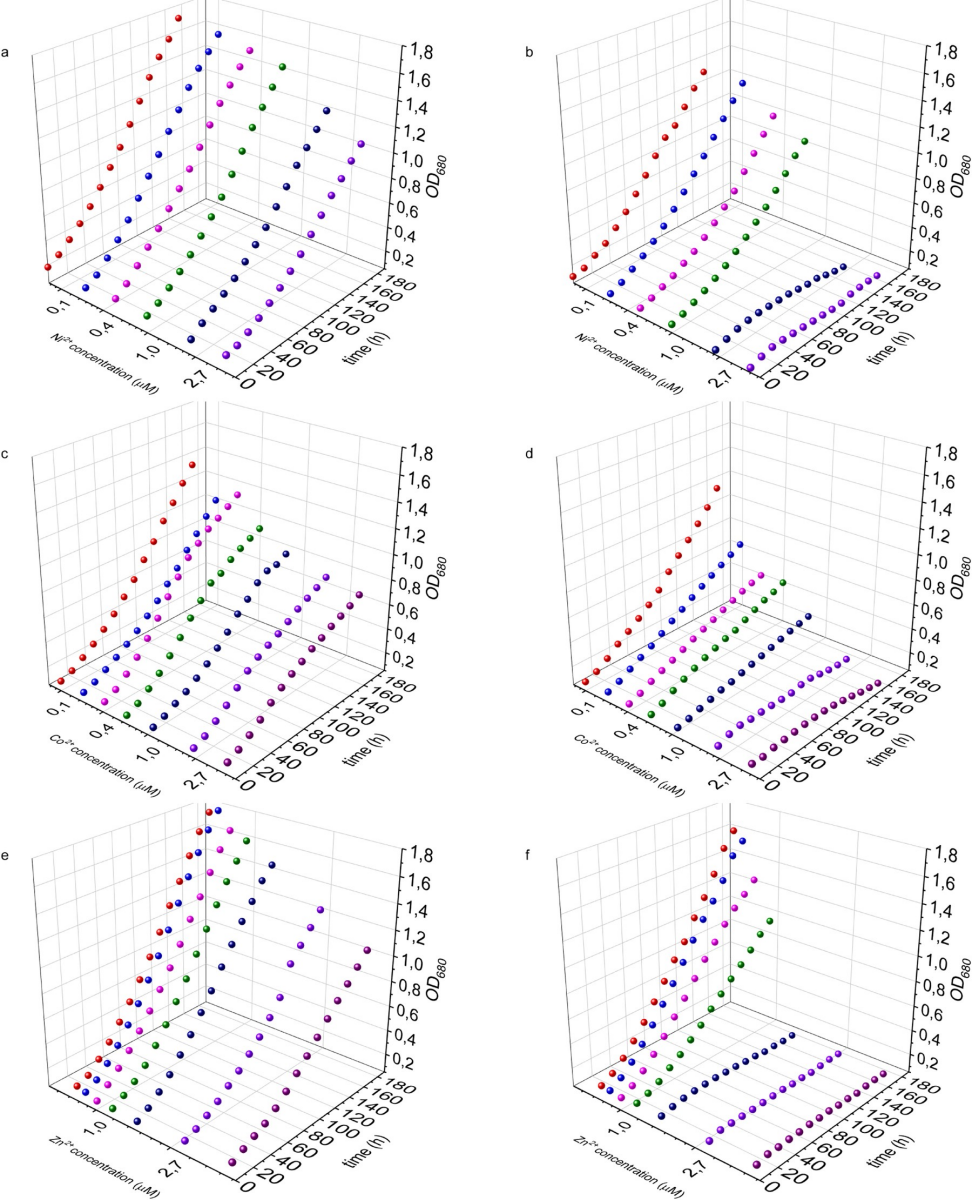
Growth inhibition. The growth curves of the WT (a, c, e) and NiCoZia (b, d, f) *Synechocystis* cell lines in different Ni^2+^ (a,b); Co^2+^ (c,d) and Zn^2+^(e,f) concentration.

First, we examined the effect of the removal of the heavy metal-specific cluster from the genome of *Synechocystis* on the growth of the cultures. To this end we treated the WT and NiCoZia cells for 98-hours with CoCl_2_; ZnSO_4_ and NiCl_2_ in 8 different concentrations (0 μM; 0.05 μM; 0.1 μM; 0.25 μM; 0.5 μM; 1 μM; 2.5 μM; 5 μM) and simultaneously measured the cells optical density (S1 File). The WT shows a faster growth rate compared to the mutant, even under control conditions without heavy metal supplementation (Fig 3).

In the investigated concentration range, increasing cobalt concentrations resulted in increasingly pronounced growth inhibition in NiCoZia strain, reaching complete growth arrest at 2.5 μM and 5 μM. In contrast, the growth inhibition was also concentration-dependent, but much less pronounced in the WT strain (Fig 3C and 3D). Similarly, zinc concentrations of 0.24 μM and up caused concentration-dependent severe growth inhibition in NiCoZia strain while this inhibition was mild and could be only observed from 1 μM Zn^2+^ in WT (Fig 3E and 3F). Lower Ni^2+^ concentrations did not cause severe growth inhibition in NiCoZia strain but also complete arrest happened at 2.5 μM and 5 μM, at which concentrations only mild growth inhibition could be observed in the WT strain (Fig 3A and 3B).

### Intracellular heavy metal content

**Fig 4:**
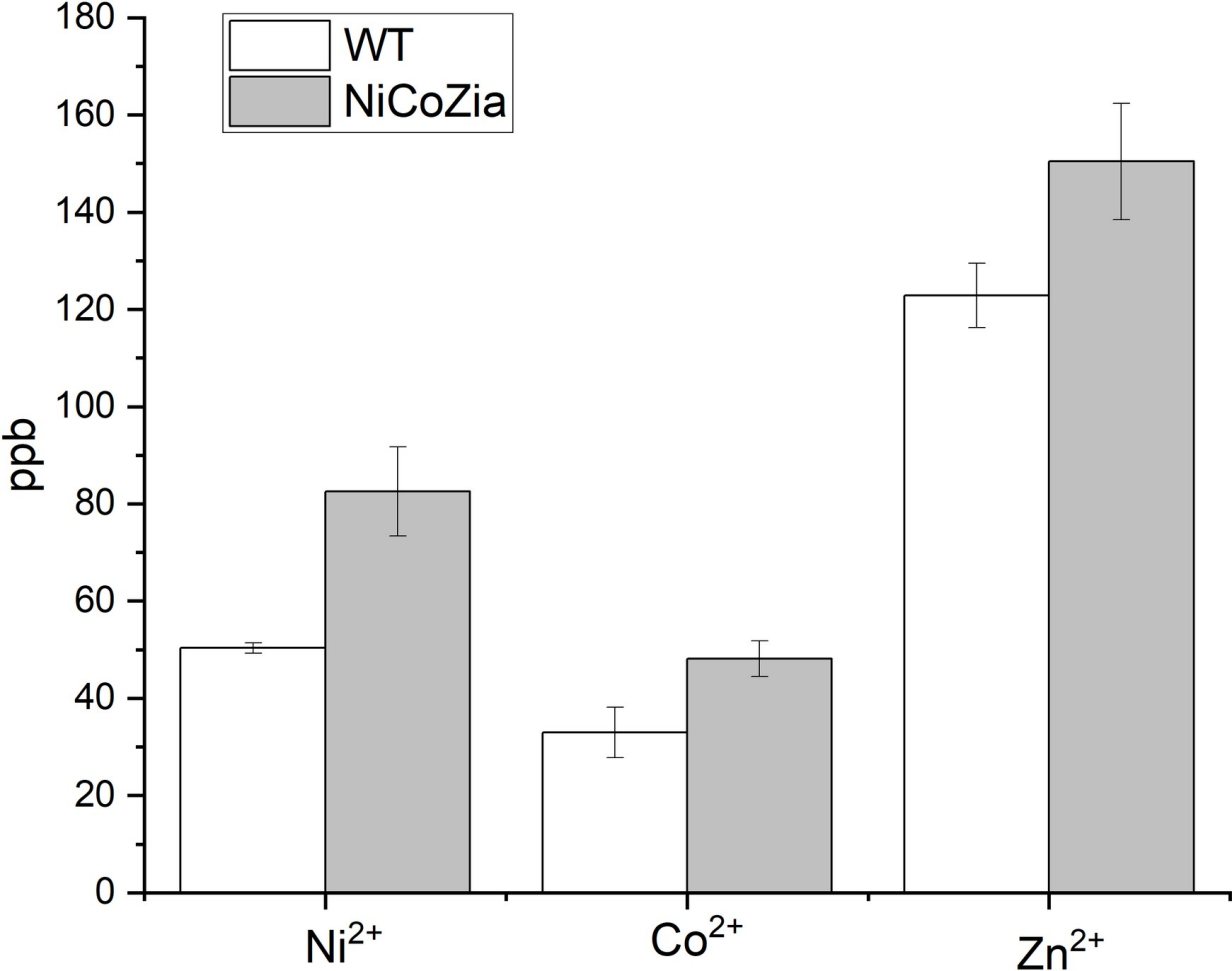
Content of the intracellular Co^2+^; Ni^2+^ and Zn^2+^ in WT and NiCoZia mutant *Synechocystis* cells. After 3-hour treatment with 5 μM heavy metal supplemented culture medium at 30 μE light intensity at 25°C temperature, the HM content was determined by ICP-MS measurement and presented in ppb (parts per billion).

Inferring from the growth curves, we concluded that in the absence of cation efflux systems encoded by the *coa*, *zia*, and *nrs* genes is likely that the intracellular heavy metal concentrations are increased as compared to the wild type. To clarify this, the intracellular heavy metal content was measured using ICP-MS in both the WT and the NiCoZia strains after 3 h long cobalt, nickel, and zinc treatments. BG-11 culture media were supplemented with 5 μM heavy metals, and then their intracellular heavy metal concentrations were compared with the untreated samples. The data (S2 File) clearly showed that the NiCoZia strain accumulates more heavy metals within cells than its wild-type counterpart does. This phenomenon was obvious with all three studied heavy metals, the largest accumulation difference observed for nickel (Fig 4), where an approximately 40% increase in intracellular nickel concentration was observed in the nickel-treated NiCoZia strain. The mutation led to an increase in about 20% Zn^2+^ and about 20% Co^2+^ content compared to WT. It is in agreement with the expectations concerning that knocked out region coded for a heavy metal exporter system.

### pILAcoaR bioreporter’s luminescent response to Co^2+^ and Zn^2+^ in WT and NiCoZia strains

**Fig 5:**
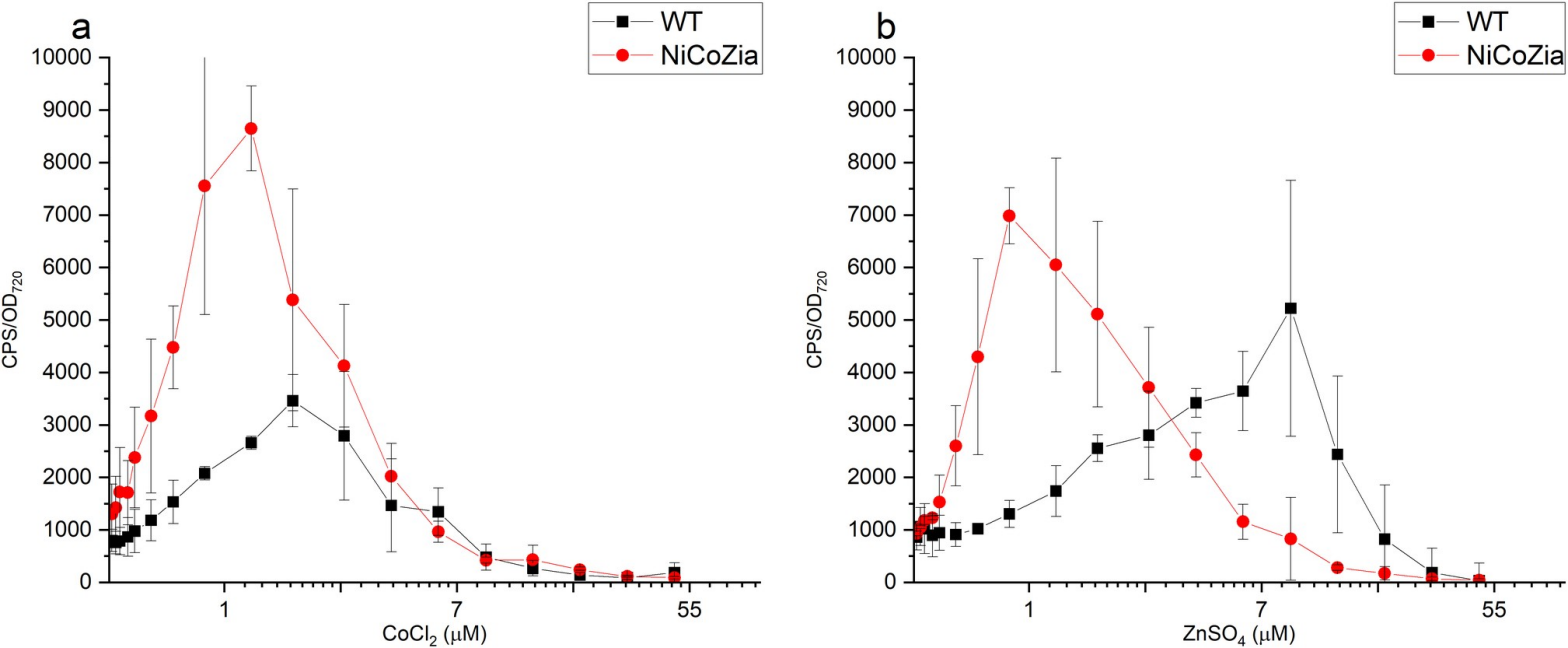
The bioluminescent response for CoCl_2_ (a) and ZnSO_4_ (b) of pILACoaR_WT and pILACoaR_NiCoZia. Cells were incubated for three hours in BG-11 medium supplemented with different concentrations of heavy metal salts before the bioluminescence was measurement. Each point represents the mean of four parallels (S3 File).

We aimed to lower the detection limits of the bioreporters, in other words, to obtain a phenotype that would be observed at higher extracellular concentrations in the wild type cyanobacterium. Hence, the luminescence of the bioluminescent pILAcoaR *Synechocystis* bioreporter strains driven by the *CoaR* promoter generated from both WT and NiCoZia strains were assessed at different cobalt or zinc concentrations to determine and compare their sensitivity and specificity for the given heavy metal. As expected, during the heavy metal treatments, the bioreporter strains with NiCoZia genomic background showed greater sensitivity. The maximum luminescence was found at 3 μM cobalt concentrations in WT, in agreement with previous studies, where the detection range was 1–3 μM [19], while the maximum luminescence response was observed at 1 μM cobalt concentration in NiCoZia. Moreover, while we found an about threefold increase in Co^2+^ sensitivity (Fig 5A) the improvement in Zn^2+^ sensitivity was about tenfold. The maximal bioluminescence response was found around 7 and 10 μM zinc concentration in WT (like earlier, Peca et al. 2008), and this value changed to 1 μM in NiCoZia (Fig 5B).

### pILAnrsR bioreporter’s luminescent response to Ni^2+^ in WT and NiCoZia strains and the effect of the orientation of the bidirectional nrs promoter on Ni^2+^ sensitivity

**Fig 6:**
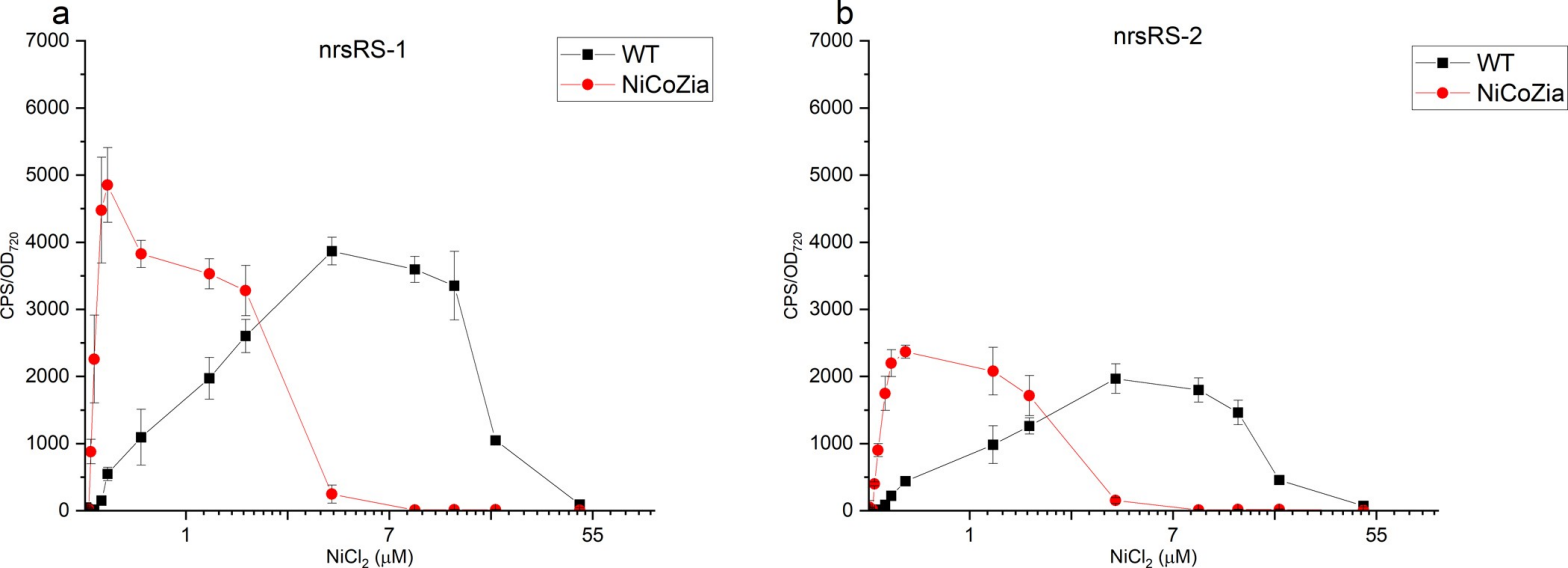
The bioluminescent responses of the pILAnrsRS constructs in the two nrsRS promoter orientations in WT and NiCoZia strains. Cells were incubated for three hours in BG-11 medium supplemented with different concentrations of (from 30 nM to 48 μM) heavy metal salt. The bioluminescence was measured as described before. Each point represents the mean of three parallels (S4 and S5 Files).

The WT strain with pILAnrsRS promoter controlled construct showed luminescence from 0.2 μM to 50 μM nickel concentration. In the NiCoZia bioreporter strain with the same construct, this range shifted by about tenfold lower concentrations compared to WT, from 0.05 μM NiCl_2_ to approximately 5 μM NiCl_2_ (Fig 6). The maximum induction changed to 0.2 μM NiCl_2_ (in the mutant) from 4 μM NiCl_2_ (observed in the WT).

Considering that these operons have bidirectional promoters, we wanted to see which direction of the promoter is to be used for the lux genes, which orientation provides better utility to our purpose. For this study, we chose the nrs promoter. We cloned the insert containing the promoter and the nrsRS gene pair in both orientations: in the first orientation (nrsRS-1) the promoter is immediately in front of the *luxA* gene, similarly to as it is oriented in the *Synechocystis* genome upstream to the *nrsB* gene. In the second, opposite orientation (nrsRS-2) *LuxA* gene is downstream of *nrsR* and *nrsS* at the other end of the insert (Fig 2A and 2B). Comparing the two constructs in WT and NiCoZia strains, it is clear that the original nrsRS-1 orientation (Fig 6A) exerts a higher luminescence induction during nickel treatment than the nrsRS-2 orientation (Fig 6B). However, the nrsRS-2 orientation is also capable of significant luminescence induction. The greater induction efficiency of nrsRS-1 orientation is beneficial in our case, nevertheless, in applications requiring lower induction levels, the opposite orientation may be more advantageous.

### pILAziaR bioreporter’s luminescent response to Zn^2+^ in WT and NiCoZia strains

**Fig 7:**
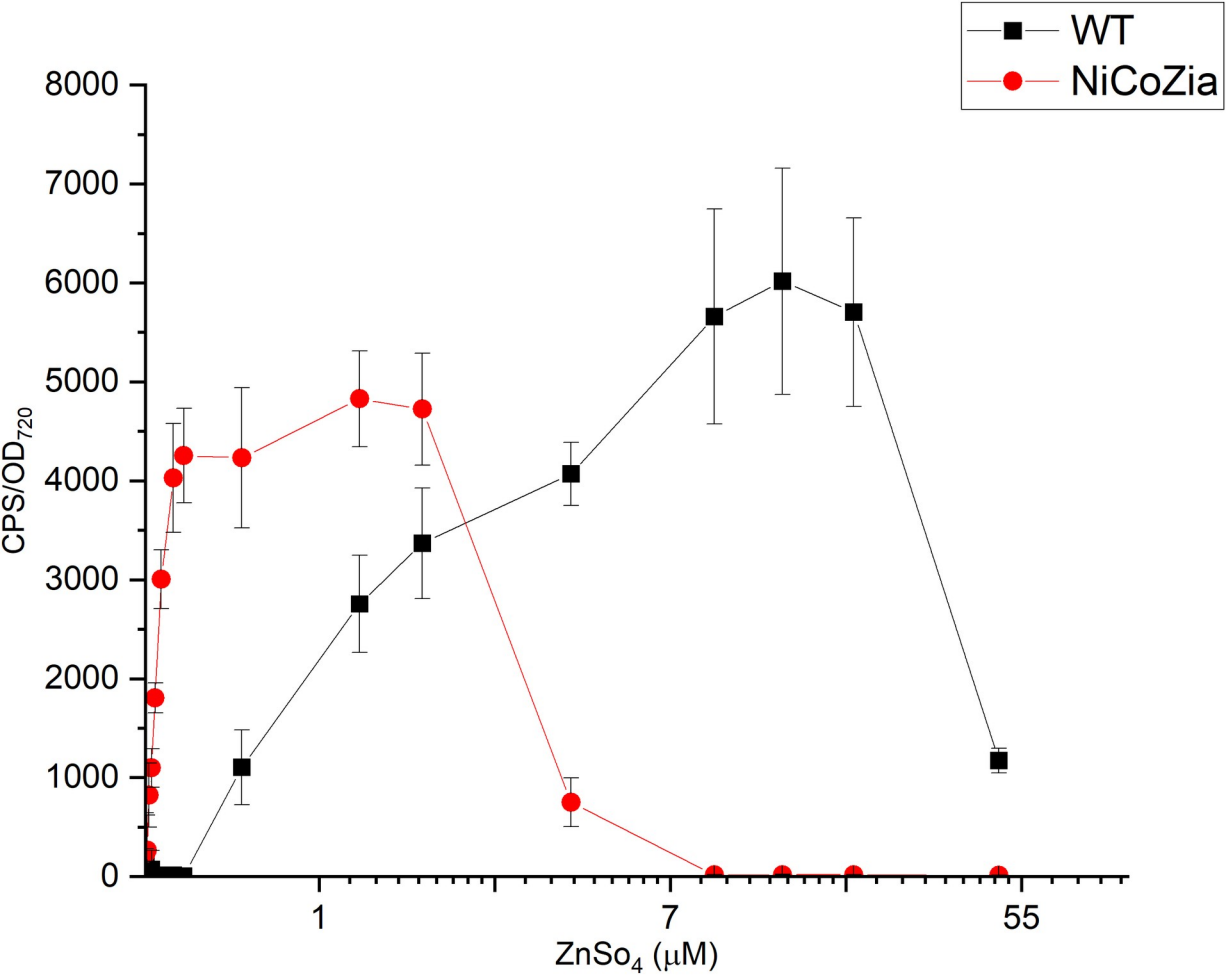
The bioluminescent response of the pILAziaR WT and pILAziaR NiCoZia for ZnSO_4_. Cells were incubated for three hours in BG-11 medium supplemented with different concentrations of (from 48 μM to 30 nM) heavy metal salts. The bioluminescence was measured as described before. Each point represents the mean of three parallels (S6 File).

Bioreporter strains with the *ziaR* promoter were treated with 0.015–50 μM ZnSO_4_ supplemented BG-11 for 3 hours, and the difference in luminescence induction (Fig 7) was similar to what we have obtained with *coaR* and *nrsRS* promoter-driven reporter genes. pILAziaR WT showed a luminescent signal in the concentration range of 0.5–50 μM ZnSO_4_ with a peak at 14 μM ZnSO_4_ treatment. This construct is more sensitive in the NiCoZia strain than in the WT background, namely, its induction can be observed from 20 nM to about 5 μM ZnSO_4_ concentration range with a peak around 1.5 μM ZnSO_4_ treatment. Thus, the construct in NiCoZia strain proved to be about 10-fold more sensitive than in WT and can be used to detect up to 0.03 μM ZnSO_4_ concentration. This value is much lower than the previous minimum detection limit of 0.5 μM ZnSO_4_ measured in WT.

## Discussion

Numerous whole-cell reporter organisms have been developed in *Synechocystis* determining a variety of metal cations in polluted soils or wastewater samples. However, in many cases, the lack of sufficient sensitivity [25] is an obstacle to their proper application (Table 2). The cellular responses to individual metal ions are dependent on their intracellular concentration, which is determined by the combined function of efflux and influx systems and ion channels. By eliminating the genes responsible for the efflux of three heavy metals we created a mutant with increased intracellular heavy metal concentration as compared to the wild type. It shows an inhibited growth compared to the wild-type strain even below the previously established [17] IC_min_ concentrations. The IC_min_ value for cobalt is 2 μM in the WT strain, whereas it is 0.1 μM CoCl_2_ in NiCoZia mutant, which means a 20-fold increased sensitivity in growth for cobalt (Fig 3D). The NiCoZia mutant was also more sensitive than the WT to zinc, showing an IC_min_ of 0.5 μM ZnSO_4_ (Fig 3F) and 1 μM NiCl_2_ concentration (Fig 3B).

**Table 2:** Genetically engineered microorganisms as Ni^2+^, Co^2+,^ and Zn^2+^ biosensors and their lowest limit of detection (LLD).

Microorganism	HM	LLD	Device	Reference
***Ralstonia eutropha***	Ni^2+^	0.1 μM	Luminometer	[26]
	Co^2+^	9 μM		
***B*. *sphaericus***	Ni^2+^	0.002 ppb (0.03 nM)	Potentiometer	[27]
***E*. *coli***	Ni^2+^	4.7 μg L^−1^ (80 nM)	Luminometer	[28]
***Microbacterium sp*. *MRS-1***	Co^2+^	200 mg L^-1^ (1.5 mM)	Microplate reader	[29]
***P*. *putida***	Zn^2+^	5 μM	Fluorimeter	[30]
***E*.*coli***	Zn^2+^	0.2 mM	Fluorimeter	[31]
***Vibrio sp*. *MM1***	Ni^2+^	6.16 mg L^−1^ (47.44 μM)	Luminometer	[32]
	Co^2+^	3 mg L^−1^ (27.93 μM)		
	Zn^2+^	0.97 mg L^−1^ (6 μM)		
***Chlorella vulgaris***	Ni^2+^	1 ppb (3.5 nM)	Conductometer	[33]
	Co^2+^	1 ppb (3.5 nM)		
	Zn^2+^	10 ppb (0.24 μM)		
***Synechocystis***	Ni^2+^	0.2 μM	Luminometer	[19]
**PCC6803**	Co^2+^	0.3 μM		
	Zn^2+^	1 μM		
***Synechocystis***	Ni^2+^	0.05 μM	Luminometer	Recent study
**PCC6803**	Co^2+^	0.2 μM		
	Zn^2+^	0.03 μM		

The increased sensitivity was the effect of the increased intracellular heavy metal concentrations resulted by the lack of the corresponding transporters, as confirmed by ICP-MS determination of the intracellular content (Fig 4). This feature could be utilized for improving the sensitivity of the specific bioreporter constructs that use heavy metal-sensitive promoter-driven expression of luminescent proteins. The luminescence measurements (Fig 5) showed that the pILAcoaR NiCoZia bioreporter strain exhibited a threefold higher sensitivity to cobalt and a 10-fold higher sensitivity for zinc than the bioreporter in the WT background. The pILAziaR NiCoZia bioreporter strain showed almost 10-fold higher sensitivity for Zn^2+^ than the WT variant (Fig 7). The pILAnrsRS NiCoZia construct also showed increased sensitivity.

Considering that we used bidirectional promoter systems, we also investigated the effect of the orientation of the cloned DNA segment. Both nrsRS-1 and nrsRS-2 oriented constructs showed concentration-dependent fluorescence induction. The nrsRS-1 orientation where the original transporters in *Synechocystis* were replaced by the reporter genes and are coded divergently from the regulator gene results in a higher level of induction and seems to be more effective than the opposite orientation (Fig 6).

The current investigation revealed that by eliminating efflux transporter genes from the cyanobacterial genome and choosing the right orientation of the bidirectional promoter system a remarkable, up to tenfold, increase in sensitivity can be obtained in constructing bioreporter strains.

It is also noteworthy that a roughly 20% increase in the internal Zn^2+^ concentration resulted in a tenfold decrease in the detection limit of the corresponding bioreporter strain. This finding shows that with limited manipulation of the genome significant improvements can be achieved, which may make the application of biosensors even more competitive and valuable method in environmental monitoring.

Biosensors for Ni^2+^, Co^2+^ or Zn^2+^ detection published in the last few years are listed in Table 2. Although their sensitivity may be remarkable, these bioanalytical tools are yet to be improved. Our current study helps to further enhance the utility of whole cell-based biosensors by facilitating the detection of lower contaminant levels in environmental samples than before.

## Supporting information

S1 File The optical density of the cultures. (XLSX)

S2 File The intracellular heavy metal concentrations in the treated and untreated cultures of WT and mutant strains. (XLSX)

S3 File Luminescence of the strains with CoaR construct upon cobalt and zinc treatment. (XLSX)

S4 File Luminescence of the strains with NrsRS-1 construct upon nickel treatment. (XLSX)

S5 File Luminescence of the strains with NrsRS-2 construct upon nickel treatment. (XLSX)

S6 File Luminescence of the strains with ZiaR construct upon zinc treatment. (XLSX)
